# Spatial and temporal distribution patterns of *Anopheles arabiensis *breeding sites in La Reunion Island - multi-year trend analysis of historical records from 1996-2009

**DOI:** 10.1186/1756-3305-4-121

**Published:** 2011-06-27

**Authors:** Louis C Gouagna, Jean-Sébastien Dehecq, Romain Girod, Sebastien Boyer, Guy Lempérière, Didier Fontenille

**Affiliations:** 1 Institut de Recherche pour le Développement (IRD), UM1-CNRS 5290-IRD 224: Maladies Infectieuses et Vecteurs - Ecologie- Génétique, Evolution et Contrôle (MIVEGEC), Montpellier - France; 2 IRD - Centre de Recherche et de Veille sur les maladies Emergentes dans l'Océan Indien (CRVOI) Sainte Clotilde, La Réunion; 3 Service de lutte anti vectorielle, Agence Régionale de Santé (ARS) Océan Indien, Saint-Denis, La Réunion; 4 Unité d'entomologie médicale, Institut Pasteur de la Guyane, Cayenne, Guyane

## Abstract

**Background:**

An often confounding facet of the dynamics of malaria vectors is the aquatic larval habitat availability and suitable conditions under which they can thrive. Here, we investigated the impact of environmental factors on the temporal and spatial distribution of larval habitats of *Anopheles arabiensis *in different locations on La Reunion Island.

**Methods:**

A retrospective examination was made from archival data which provided the complete enumeration of *An. arabiensis *breeding habitats in three distinct geographic zones - extending North-east, West and South of the island over 14 years, from January 1996 to December 2009. Data on the occurrence and the number of active larval habitats at each of a total of 4376 adjacent ellipsoid grid cells (216,506 square meters each) were used (1) to provide the geographic extent of breeding site availability from year to year and (2) to analyze associations with prevailing environmental factors, habitat types, and locations.

**Results:**

*Anopheles arabiensis *utilized a spectrum of man-made and natural aquatic habitats, most of which were concentrated primarily in the rock pools located in ravines and river fringes, and also in the large littoral marshes and within the irrigated agricultural zones. The numbers of breeding site per sampling grid differed significantly in different parts of the island. In contrast to an originally more widespread distribution across the island in the 1950s, detailed geographic analyses of the data obtained in the period extending from 1996-2009 showed an intriguing clustered distribution of active breeding sites in three discontinuous geographic zones, in which aquatic habitats availability fluctuates with the season and year. Seasonality in the prevalence of anopheles breeding sites suggests significant responsiveness to climatic factors.

**Conclusions:**

The observed retreat of *An. arabiensis *distribution range to lower altitudinal zones (< 400 m) and the upward shift in the most remote littoral areas in the northeast and southwest regions suggest the possible influence of biogeographic factors, changes in land use and control operations. The results of this study would allow for a more rational implementation of control strategies across the island.

## Background

In La Reunion Island, malaria has gradually disappeared since the 50s [1,2], but this feature remains fragile considering the unfavourable epidemiological situation of the disease in the majority of the countries or territories of the southwest area of Indian Ocean. The development of tourist exchanges between La Reunion and other neighbouring islands, where endemic malaria still persists, may favour regular introduction of imported malaria cases on the island [3,4]. Among the factors responsible for this threat are the climatic and ecological characteristics that favour the persistence of *Anopheles arabiensis*, a member of the *An. gambiae *complex [5]. A few local cases of *Plasmodium falciparum *malaria have been reported three times in 2000, 2005 and 2006 (The French Ministry of Health - ARS-Indian Ocean, unpublished data). This attests to the vulnerability of the island with regard to a malaria transmission risk. Such threat of the resurgence of malaria (i.e. the presence of malaria vectors and the regular introduction of infected patients) in this tropical island is a major public-health issue [6]. This calls for an intensive planning effort to prepare not only for the possible re-emergence of malaria, but for a long-term comprehensive approach to preventing mosquito-borne diseases. From the entomological perspective, no previous studies allow direct inferences about the risk of re-emergence of malaria in La Reunion.

An often confounding facet of the dynamics of malaria vectors is the aquatic larval habitat availability and conditions under which they can thrive. In 1950s, Hamon reported a coarse scale distribution of breeding sites of *An. gambiae s.l*. across different ecological systems on the island [7]. This historical distribution remained primarily littoral with penetration inside the mainland in the northwest and southwest regions. From 1970s onwards, the occurrence and distribution of larval habitats in the dense dwelling zones was highly focal, presumably because of urban development and subsequent land-cover changes, good drainage of the urban areas which limits open lands available for the aquatic habitats and intensive larval control efforts [5]. Furthermore, the data obtained during the period from 1985 to 1987 confirmed the persistence and wide range distribution of many breeding habitats in the littoral fringes in the north-eastern, the western zones and south-western zones [8].

Control campaigns against mosquito larvae with targeted pesticide use began in 1950s and continued on a regular basis until 1990 in the absence of detailed entomological knowledge [9-11]. These campaigns have not only focused attention on *Anopheles *niches but also on the *Aedes sp*. breeding sites constituted by artificial waste storages [11,12]. However, the use of insecticides (e.g. DDT and temephos) has being progressively marked by direct human health concerns about food and groundwater contamination and negative impact on the biodiversity of friendly insects [13]. The contemporary strategy emphasizes the use of *Bacillus thuringiensis israelensis *serotype H14 (*Bti*). The behaviour of the local *Anopheles *populations, more exophilic than endophilic, even more zoophilic than anthropophilic [14], is one of the reasons why vector control plan relies primarily on the larval control. The major advantages of the biolarvicides are an easy application in the field, its safety to the environment, human beings, animals and other non-target organisms [15]. However, *An. arabiensis *is being maintained on La Reunion in spite of regular vector control actions that target mainly larval habitats [9,10]. This has generated the need for new alternative strategies to overcome the limitations of the larval source management strategy to control malaria vector on the Island. La Reunion is currently being considered as a potential site to undertake field trials using innovative vector control techniques, such as the Sterile Insect Technique (SIT) [16,17]. On this island, *An. arabiensis *is a logical target for testing the efficiency of potential area-wide releases of sterile male mosquitoes in the field. Information about optimal spatial and temporal environmental conditions that sustain *Anopheles *populations in the island is needed.

The objective of this study was to define the habitat ranges of *An. arabiensis *in La Reunion Island, and to determine whether the presence or absence of their larval habitats could be predicted based upon environmental variables at three distinctive regions of the island. Historical data over the recent decades provide new insights on the temporal shifts in the range distribution of active *An. arabiensis *breeding habitats from 1996 to 2009. This information could be useful for decision making during control operations aiming at preventing the re-emergence of malaria in the island.

## Materials and methods

### Study area

La Reunion is a volcanic island located in the southern hemisphere at latitude 21°55' South and longitude 55°30' East, in the South-West of the Indian Ocean, 800 km from the east of Madagascar and 200 km from Mauritius. This island has a surface of 2,512 km² and about 810,000 inhabitants. The human population density ranges from 25 to 500 inhabitants per km^2 ^distributed in small nuclei. Topographically, the island area consists of high-elevation, mountainous zones (only 25.5% of La Reunion falls below 500 meters), foothill plateaus and artificial or highly modified costal fringes. Thirty three percent of the island is covered by a natural surface characterized by sharp reliefs and abrupt wooded areas, large unoccupied volcanic areas and beaches. Another important aspect of the island in connection with *Anopheles *ecology is the existence of a dozen permanent rivers though at very irregular flow and a large network of more than 100 torrents (called ravines) which experience sporadic water flow only a few days per annum. These ravines (totalling 10, 650 km) retain water after each raining event in each of the thousand rock pools of their bed. The general orientation of the island has created three contrasted climatic areas: a dry western area under the wind, a wet area on the wind at the North-East and a third intermediate area on the mountain relief of the South and the center of the island. These anemometric phenomena, at microclimatic scale, are extremely complex but their knowledge is of interest to understand the ecology of mosquitoes. Climate is typically that of a tropical region but is largely under oceanic influences. It is characterized by a hot rainy season which usually lasts from November to April and a dry winter which begins in May and ends in October.

La Reunion has a small mosquito fauna consisting of only 12 species of mosquitoes. *Anopheles arabiensis *is the only member of the *An. gambiae *complex which has been reported in the peri-urban habitats and along coastal areas [5,7,8]. Its bionomics have been studied to some extent during the 1950s and late 1990s [7,14,18]. *Anopheles coustani *also occurs in La Reunion. An account of previous epidemiological evidence collected in La Reunion, together with the information from the neighbouring islands, indicates that this exophilic species is a prolific man biter but is non-vector of malaria parasites [5,7]. Ten other mosquitoes species belonging to *Aedes, Culex, Orthopodomyia *and *Lutzia *genera have also been found in a wide range of habitat types [3,5,7]. Here, the focus is on *An. arabiensis *since the presence of this efficient malaria vector places the island at risk of malaria re-emergence and the need to prioritise areas for larval control remains greatest.

### Data collection

**Figure 1:**
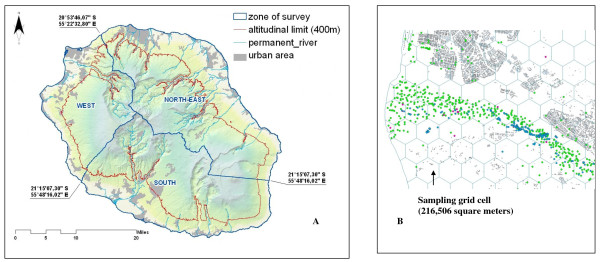
***Anopheles arabiensis *larval inspection sites in La Reunion from 1996 to 2009**. The figure shows the typical area which was mapped and serves to illustrate the land division into sampling grid cells during routine larval surveys. Box A shows the location of study sites in La Reunion. Red line indicates the altitudinal limit of each sampling zone, blue lines shows study region borders in western, southern and north-eastern region. Box B indicates an example of sampling grids from 1996 to 2009 in the north-eastern study area (as in western and southern zone).

An analysis of original records over 14 years (from January 1996 to December 2009) was carried out. The computerized dataset was retrieved retrospectively from official data repositories at the local mosquito control agency. These unpublished backups provide the complete enumeration of mosquito breeding sites identified by field teams over the past 50 years, thus allowing an historical view of temporal patterns of presence or absence (irrespective of larval density) of *An arabiensis *breeding sites at different geographic levels in La Reunion [7,8]. Only data which were acquired during continuous monthly surveillance over the period ranging from 1996 to 2009 were considered depending on the availability of environmental datasets. Three different zones - extending North-East, West and South of the island were selected (Figure 1A). These zones coincide with distinct spatial units of habitats characterized by highly contrasted climatic features (wind exposure, extent and pattern of seasonal change in temperature and rainfall.

### Identification and characterization of breeding sites

During field visits, all open water bodies (excluding running water and water in containers in houses) containing immature stages of anopheline mosquitoes were first counted. Location and elevation of each active habitat type was recorded using a hand-held global positioning system (Garmin Inc.). These field surveys conducted over 14 years each month allowed quantifying a series of environmental elements of suspected relevance to *An. arabiensis *ecology such as vegetation and hydrological systems. Habitat type was categorized into stream, small and large drain channels, swamps, rock pools, puddles, burrow pits, rain pools, stream bed pools, wet meadows, artificial holes, concrete hole, and artificial containers. These were subdivided into permanent and temporary pools, referring to any discrete body of water that was likely to remain inundated for at least one month and less than one week, respectively. On each occasion, a voucher collection of larvae found in each breeding site was assembled as a standard reference for species identification. Larvae samples recovered from active breeding sites were identified to species by morphological criteria. From 2004 to 2009, species-specific polymerase chain reaction (PCR) was implemented to confirm the results of previous morphologic identifications. The data obtained during the period that spans from 1996 to 2009 was considered because (1) the breeding sites were monitored continuously using standardized larval sampling methods [8], and (2) the existing database provide a detailed enumeration of environmental variables necessary for this study.

### Selection and categorization of land-use sites

We used a geographic information system (ArcView v9.3, ESRI, CA-USA) to define ellipsoid grid cells as sampling units of approximately 216,506 square meters each (Figure 1B). This provided a more comprehensive framework for the interpretation of the variation in the geographic distribution range of active breeding sites. With the assumption that distribution of larval habitats was homogeneous among the different grid cells, random samples of adjacent sentinel grids at each of the 3 zones were selected. In the corresponding study zones, water bodies were localised in non-contiguous isolated areas (Figure 1). Many of these are associated with swamps, rivers, and other natural water bodies which in the last decade have provided permanent breeding sites for malaria vectors. The elevation limit laying within 0 - 400 m above sea level was chosen because it best described the range of *Anopheles *breeding sites in La Reunion [8], including historical foci with larval habitats [7], and for which the larval surveys have been performed with identification of the collected species. The remaining zones at the altitude above 400 m were not sampled because the territory is very abrupt and inaccessible since the landscape is highly fragmented into forests and volcanic mountains.

An important part of the current analysis was based on the assessment of the quality of existing data on the relative abundance (presence and absence) of *Anopheles*-positive aquatic habitats per month during three periods of 1996-1997, 2002-2003, and 2007-2009. Land-cover in these absence or presence locations was classified into categories relevant to mosquito habitats, i.e. irrigated croplands, rain-fed flooding or croplands, urban areas, forests and coastal zones. Land-use was also classified into two broad categories namely natural, essentially consisting of areas with typical natural vegetation cover with little or no human interference and artificial areas with profound environmental alterations due to human uses such as agriculture and development areas. The artificial land-use types at each of the study zones were further subdivided into elements of clearly negative influence for the presence of *Anopheles *breeding sites such as roadway infrastructures and residential area. The nearest distance between human habitations, water bodies and farm edges was also recorded.

### Climate

Climate data was obtained from two local meteorological stations per zone and located near the aquatic habitats initially selected for sampling at *Le Port *and *Pointe des 3 Bassins *for the western zone, at *Saint Benoit *and *Gillot-Airport *for the north-eastern area and at *St. Joseph *and *Pierrefond *for the south, respectively. The data, available from the meteorological national database built by Meteo-France, spans from 1996-2009 and describes rainfall estimates (monthly total amount of rainfall, the number of rainy days, daily max rainfall, monthly mean/max/min relative humidity, monthly mean/max/min temperature, and max wind speed). Records from more than one weather station from each zone were used and assuming that climatologic values were repeatable among weather stations within zones, we averaged the values among stations to obtain one entry that was an approximation of the data within each zone.

### Statistical analyses

The sets of data in the form of presence or absence and the number of the focal *Anopheles *breeding sites in a sample of grid cells were combined to produce geographic distribution range maps for *An. arabiensis *habitats. The first step towards developing such tools was to examine relationships between the frequency of larval habitats and environmental conditions, including aquatic habitat type, physical habitat conditions, and surrounding land use. Depending on the year, a variety of graphical representations were specifically developed using the built-in GIS software functions (Arc-Info GIS 9.3 grid module, ESRI Inc) to model the distribution of the variables within each study zone. The main inputs consisted of geographically referenced boundaries of reporting units at the highest possible resolution. Layers of urban areas, infrastructures, river systems and topography produced by the National Geographic Institute have been used as background information to create the variables used. A total of 4376 grid cells (referenced on the UTM projection system, international ellipsoid) located less than 400 m of altitude were used for the layers and have been grouped into different types according to their significance. Visual examination and comparison of the various maps guided by statistical evaluation was made to help rank the relative importance of the *An. arabiensis *breeding sites in an area with regards to prevailing local conditions. The importance of rainfall (monthly, annual precipitation) and temperature (minimum and maximum) in each year concurrent with larval sampling was investigated. Their temporal patterns over the whole study period were decomposed into an additive series of different seasonal frequencies. We also tested whether data for altitude, pattern of household density for any sampling zones may produce enough variation throughout the study period to result in measurable differences in the distribution of breeding habitats that could be related to these environmental gradients. The distances from each breeding site boundary to the nearest household were generated using the distance tool within the ArcView software.

The General linear models (GLM) and Pearson's chi-square test, for continuous and categorical variables, respectively, were used to compare the group means of environmental variables for larval habitats between the three study zones and year. Relationships between the presence-absence of Anopheles breeding habitats within each sampling unit and the selected environmental variables in individual zone were explored using logistic regression analyses, while the associations further between the mean counts of positive *Anopheles *larval habitats with climatic variables and environmental parameters were examined using GLM. All continuous variables were subjected to log transformation before further analysis. The statistical significance was set 0.05 unless otherwise stated. All statistical analyses were conducted both with Excel and the statistical software package SPSS (version 18.1 for window).

## Results

A monthly surveillance was conducted on a total of 4,376 discrete grid cells located in high priority zones for larval control operations - with an over-sampling of those areas where there were few larval sites during the study period. Repeated sampling from 1996 to 2009 allowed providing quantitative or qualitative descriptions of *An. arabiensis *breeding sites in the northeast (on a maximum of 1,935 grid cells/year), south (1,462 grid cells/year) and west (979 grid cells/year) zones, respectively. The numbers of grid cells sampled within each of the three zones were similar across the three periods (see Table 1). Therefore, the sample size provides a reasonable basis for comparing the temporal and spatial pattern of breeding site occurrence within the corresponding areas. By morphological identification and PCR characterization, larvae recovered at active breeding sites were composed of *An. arabiensis *although a few of them contained *An. coustani *larvae. The immature stages of the latter species were mainly encountered in small brackish pools with abundant weeds and vegetation. Because its distribution was captured poorly by field surveys, the emphasis of the following sections is on *An. arabiensis *habitats in regards to their spatial and temporal changes.

**Table 1:** The proportion of occurrence and average number of *Anopheles arabiensis *breeding sites in sentinel grid cells across different ecological zones in La Reunion Island from 1996 to 2009.

Studied zone	Sampling period	Numbers of grids sampled	Number of positive grid cells (%)	Total number of breeding sites	Mean (M) number of breeding sites (SEM)	Observed maximum number	Variance (V)	Dispersion (V/M)
NORTHEAST	1997	1972	136 (6.90)	99	1.37 (0.07)	6	0.60	0.44
	2003	2129	305 (14.33)	109	2.80 (0.30)	17	10.34	3.69
	2009	2317	558 (24.08)	176	3.17 (0.32)	27	18.32	5.78
SOUTH	1997	1470	34 (2.31)	26	1.31 (0.12)	3	0.38	0.29
	2003	1552	150 (9.66)	60	2.50 (0.22)	10	3.13	1.25
	2009	1616	256 (15.84)	102	2.51 (0.28)	16	8.11	3.23
WEST	1997	1164	285 (24.48)	100	2.85 (0.26)	16	7.01	2.46
	2003	1003	52 (5.18)	31	1.68 (0.17)	4	0.95	0.57
	2009	1185	246 (20.76)	40	6.15 (1.84)	67	136.02	22.11

Prevalences (%) are given for the total numbers of grid cells within each zone that did have at least one larval habitat in the specified year. Total and mean numbers of breeding sites per individual grid cell are also displayed. Data is presented separately for each of the 3 study periods

### Description of the observed *An. arabiensis *larval habitats

**Figure 2:**
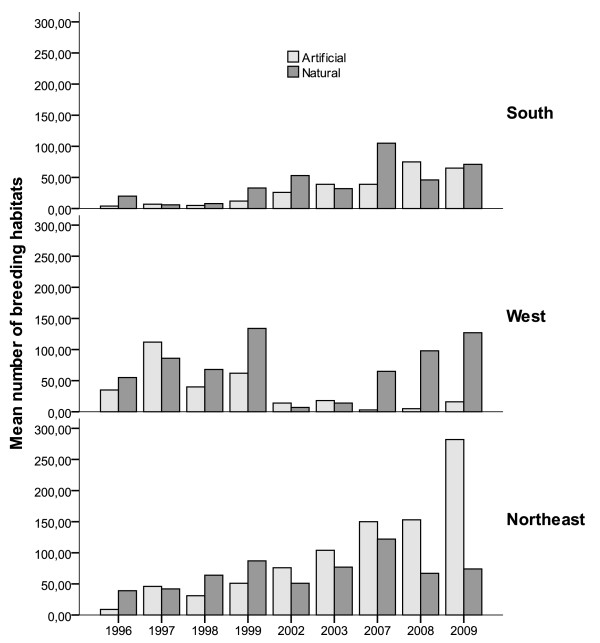
**Yearly variations in the prevalence of different *An. arabiensis *breeding typology within sentinel grid cells in distinct eco-geographic zones in La Reunion Island from 1996 to 2009**.

At the level of individual habitat typology, occurrence of larvae was documented predominantly in permanent ponds, but this characteristic was noticeable in the northeast and west areas where only 48.9% and 27.1% of the all observed breeding sites were temporal, respectively (Table 2). Restricting analyses to the positive grid cells (where breeding sites were detected), *An. arabiensis *larvae were found mainly in natural habitats, i.e. fringes of the perennial rivers whose flow is generally very irregular, rock pools of the many ravines with temporary flow, ponds and residual puddles in the inhabited areas (ground holes). This characteristic was markedly observed in the western zone where 31.2 - 86.7% of the habitats were artificial consisting of roadside ditches, water reservoirs, water channels, roadside ditches or puddles pools due to the irrigation in the agricultural zones, and artificial containers. Within each studied zone, the average numbers of the distinct habitat types were relatively unchanged during the period from 1996-1997 and 2002-2003. During the transition from the 1^st ^(1996-1997) to the 3^rd ^period (2008-2009) the average numbers of natural and artificial habitats increased significantly in both the north-east (natural: 37.9 versus 62.1%; artificial: 11.9% versus 88.1%) and southern (natural: 13.1% versus 86.9%; artificial: 10.6% versus 89.4%) regions (Figure 2). Furthermore in the western zone, there was a decrease in the total proportion of artificial habitats (86.7% versus 13.3%) and increase in natural habitats (39.7% versus 60.3%) from the beginning to the end of the study period. This inter-annual fluctuation trend in the occurrence of (both natural and artificial) larval habitats was observed, with significant increase over time in the northeast (Pearson's χ^2 ^= 1086.1, df = 4, *p *< 0.001), southern (χ^2 ^= 440.6, df = 4, *p *< 0.001) and western (χ^2 ^= 458.9, df = 4, *p *< 0.001) zone. Subsequently throughout the study period, the north-eastern area exhibited significantly higher group means for any habitat types compared to the southern (OR = 2.86, 95% CI [3.06 - 5.01], *p *< 0.001) and the western zone (OR = 3.91, 95% CI [2.19 - 3.73], *p *< 0.001).

**Table 2:** Key parameters measured in the aquatic habitats that contained *An. arabiensis *larvae in La Reunion.

	North-East		West		South	
	
Characteristics & type of breeding sites	Number breeding sites	% within zone	Number breeding sites	% within zone	Number breeding sites	% within zone
River-fringes	16	1.6	104	17.8	35	8.0
Water Channel	23	2.3	9	1.5	12	2.7
Swamps	2	0.2	0	0	0	0
Tyre tracks	428	42.9	138	23.7	48	10.9
Residual Puddles	100	10.0	13	19.0	130	29.5
Artificial containers	12	1.2	13	2.2	23	5.2
Water reservoir	6	0.6	10	1.7	3	0.7
Rock pool	107	10.7	146	25.0	39	8.9
Others	303	30.4	52	8.9	150	34.1
Total	997	-	583	-	440	-
**Habitat characteristics**						
Permanent	206	20.7	373	64.0	130	29.5
Temporal	488	48.9	158	27.1	160	36.4
Semi-permanent	303	30.4	52	8.9	150	34.1
**General surrounding**						
Household density (%)*	38.5 (1.8)	-	30.4 (0.2)	-	53.8 (0.3)	-
Mean Distance (meter) to the nearest inhabited house (range)	209.2 (2.5 - 6,000)		198.4 (4.1 - 896.5)		181.1 (5,000 - 19,000)	
**Mean Altitude (meter) **(Range)	46.7 (0 - 342.7)		80.9 (2.4 - 393)		85.5 (1.3 - 372.7)	

* Considering only positive grid cells, values represent means (Standard Error of Mean)

**Figure 3:**
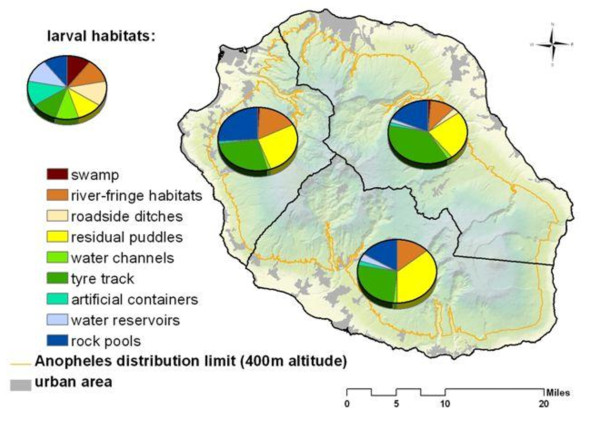
**Distribution of *An. arabiensis *breeding site categories in distinct eco-geographic zones in La Reunion Island from 1996 to 2009**.

The composition of breeding site types was broadly similar across the three zones (Figure 3), but their annual numbers during the study period differed significantly in average in the north-eastern (χ^2 ^= 1068.1 df = 16, *p *< 0.001), western (χ^2 ^= 732.6, df = 14, *p *= 0.004) and southern (χ^2 ^= 517.1, df = 14, *p *< 0.001) zones. Irrespective of the period of survey, tyre tracks dominated among the breeding sites recorded in the north-east (proportion of occurrence: 42.9%), western (90.5%) and southern zone (31.3%). It was noted that the bulk of these breeding sites were represented in decreasing order by ponds, tyre tracks, rock pools, habitats located in the river fringes and extensive gully systems that furrow the entire Island, and road side ditches. Within the three zones however, others habitat types including shadow residual puddles (mainly from rain fed), water reservoir, water channels and artificial containers represented a small proportion of the total breeding sites type recorded (Table 2). The southern zone had the narrowest arrays of breeding sites as compared to the types recorded in other zones.

### Annual prevalence and geographic distribution of *An. arabiensis *breeding sites from 1996 to 2009

**Figure 4:**
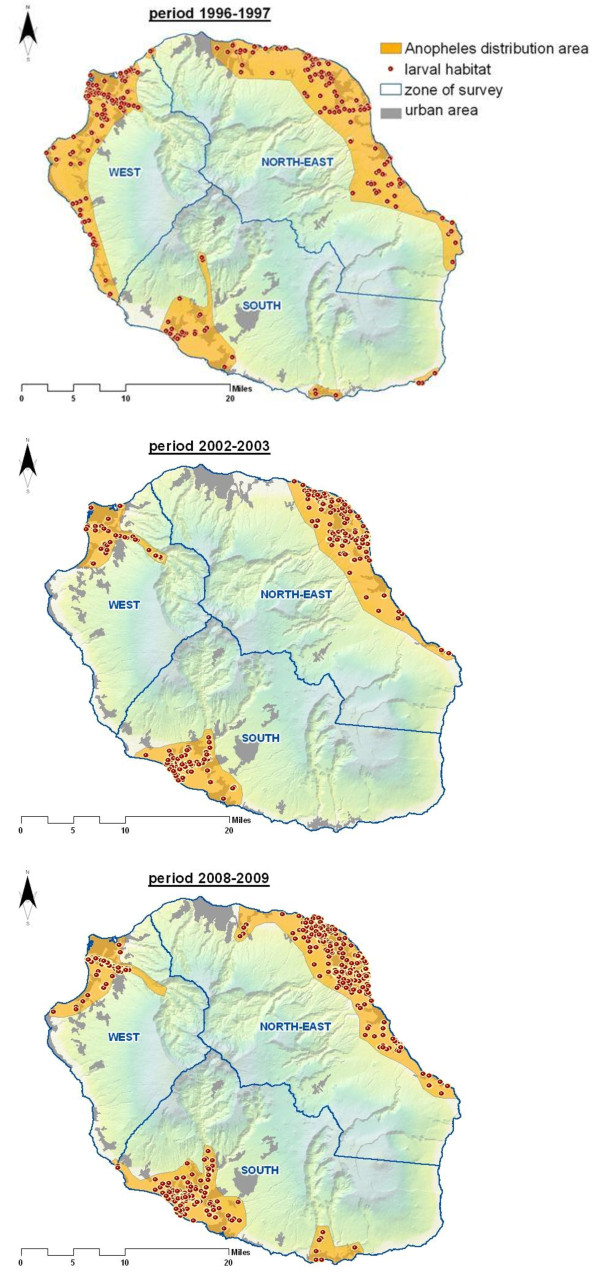
**Temporal trends in the geographic range of *An. arabiensis *breeding sites in the three study zones**. Monthly surveys were carried out at each zone mostly in urban and peri-urban habitats near the coast or at altitude ≤ 400 meters. Compared to the previous records (Figure 5 below) these figures show a clear temporal trend in the reduction of the distribution range of *An. arabiensis *breeding habitats. They persist in certain discrete foci in western, northeast and south where however, absolutely high numbers of larval habitats were observed in 2008-2009, compared to previous years.

The geographical distribution of *An. arabiensis *larvae habitats throughout the three study periods considered - 1996-1997, 2002-2003 and 2008-2009 - is presented in Figure 4A-C where each point within any sampling unit corresponded to a single habitat. The three studied periods were compared for the percentage of occurrence and density of breeding habitats within and between the zones (North-East, West and South). In spite of the discrete spatial distribution pattern of *An. arabiensis *positive habitats, the prevalence of positive grid cells increased by 75% from the 1^st ^(136/1935) to the 3^rd ^period (558/1935) at the north-eastern zone and by 86% (34/1462 to 256/1462) at the southern zone. In parallel, a slight decrease (~13%) of the prevalence of grids with productive larval habitats was recorded at the western zone from 1996-1997 (285/979) to the 2008-2009 (246/979). A logistic regression indicated that there was a significant zone effect (Wald χ^2 ^= 16.87, *p *< 0.001) or sampling grid within each zone (χ^2 ^= 7.79, *p *= 0.005), year (χ^2 ^= 32.72, *p *< 0.001) and season (χ^2 ^= 3.62, *p *= 0.06) on the probability of a breeding site occurrence (Figure 4). In general the estimated risk of occurrence of active breeding sites per grid cell remained significantly lower in the southern zone than that from any other zone in 1996-1997 (OR = 0.07; 95%CI [0.05 - 0.10]; *p *< 0.001), but also in 2002-2003 (OR = 1.95; 95%CI [1.41 - 2.71]; *p *< 0.001) and 2008-2009 (OR = 0.71; 95% CI [0.59 - 0.87]; *p *= 0.001).

The number of larval habitats varied significantly among positive grid cells (F_1, 13106 _= 6.25, *p *< 0.001). There were no systematic variations of the average numbers of breeding sites per grid cells between the three zones (F_2, 4001 _= 0.31, *p *= 0.74), but the magnitude of the change in the numbers of breeding sites between zones often varied consistently between the three periods (zone × sampling period: F_4,13117 _= 23.91, *p <*0.001). Adjusting for multiple pairwise comparisons in the mean number of breeding sites between the three periods, the mean difference was significant at the 0.01 level for each pair except for the transition between the first (1996-1997) and the third (2008-2009) period. In addition, compared to the 1996-1997, the distribution of counts of breeding sites per zone appears highly aggregated throughout the period from 2008 to 2009, being more dispersed within the western (Variance to Mean ratio: 2.46 vs. 22.11) and north-eastern (V/M = 0.44 vs. 5.78) zones than in the south (V/M = 0.29 vs. 3.23), indicating that habitats of the different sampled grid cells within each zone were not equally suitable for breeding habitat occurrence.

**Figure 5:**
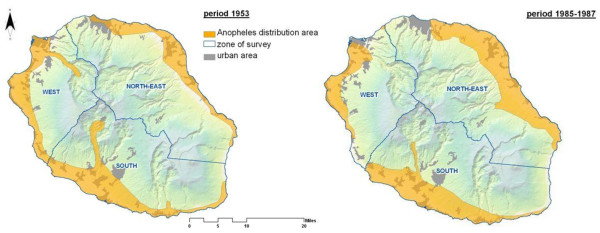
**Historic maps showing the spatial distribution of *An. gambiae s.l *in 1953 (A: Source, Hamon, 1953) and for the period 1985-1987 (B: source, Regional Health Agency) in La Reunion island**.

Analyses of the temporal and spatial changes in breeding habitat availability indicated a distribution pattern that appeared much less broad from 1985 to 1987, in contrast to an originally more widespread distribution across the island in the 1950s (Figure 5). Thereafter, *An. arabiensis *seems to have permanently disappeared in some zones, but persist in three discontinuous geographic zones (Figure 4A-B). For instance, detailed geographic analyses of the 1996-2003 records show an intriguing distribution of active breeding sites along the southwest and northeast costal lines, in which aquatic habitats availability fluctuates with the season and year. Compared to 1996-1997 and the period ranging from 2002 to 2003, the frequency and area positive for *Anopheles *breeding sites increased approximately two-fold during the 2008-2009 period, especially in the north-eastern areas reporting higher proportion of breeding sites occurrence, compared to the southern and western zone. 75% of the breeding sites recorded in 2008-2009, some of which are of extremely ephemeral nature, occurred along the large flat littoral zones situated at altitude less than 100 meters above sea level, with high precipitation rate, extensive irrigation practices and scattered local housing.

### Influence of climatic and environmental factors on larval habitats occurrence

#### Climatic data

Year-dependent variations in the number of rainy days (F_2, 109 _= 102.6, p < 0.001) and the mean precipitation (F_2, 109 _= 39.7, p < 0.001) were more pronounced in the north-eastern zone (Mean + SEM rainy days per year: 14.3 + 0.47; annual precipitation: 236.8 + 14.07 mm) (4.12 and 2.78 times higher) than it is in the Southern zone (rainy days: 8.7 + 0.78; precipitation: 113.3 + 22.9 mm), while the western region has experienced irregular periods of dryness and lower mean of precipitation every year (mean rainy days: 3.3 + 0.86; precipitation: 53;6 + 14.07 mm). A planned comparison in the mean monthly temperature (both minimum and maximum) between the north-eastern, western and southern zones during the recent decade (i.e. 1996-2009) showed no significant inter- annual differences. The relative humidity varies on the littoral from 70% to 98% in hot and rainy season and from 50% to 80% in fresh and dry season.

### Relationships between climatic/non-climatic variables and breeding site distribution

Some disparities in individual habitat types according to the season of each year were observed at all the three zones (OR for habitat types occurrence in the rainy versus dry season = 34.2, 95% CI [11.43 - 102.49], *p *< 0.001), with the end of the rainy season (April) coinciding with the greater percentage of occurrence of swamps, ponds and residual paddles and rock pool. In contrast during the driest months of each year (July - October) the relative variety of breeding site types declined and the small temporal ponds, residual puddles in form of rain-fed pools, water channels, and clusters of tyre tracks were absent within individual sampling zones. There was no relationship between the proportions of positive grid cells and climatic variables at any of the study zones. However in almost all the three study periods, climatic variables such as rainfall (F_1, 13106 _= 8.3, *p *= 0.002), minimum (F_1, 13106 _= 6.3, *p *= 0.04) and maximum (F_1, 13106 _= 6.7, *p *= 0.02) temperature, showed a strong influence on the geographical variation of the mean number of breeding sites. The greatest rate of increase of *An. arabiensis *breeding site numbers appeared to coincide with the onset and during the period following the end, rather than the peak, of the rainy season. Indeed, irrespective of the geographical zone and survey periods the average number of breeding larval habitats within each positive grid cells varied from one season to another (GLM, Zone × Period × Season interactions: F_5, 13106 _= 7.8, *p *= 0.002). The counts were significantly lower at the end of the dry season (November December), and increased in January and February and reach their maximum at the end of the hot and rainy season in March and April. In contrast to temperature, mean humidity or any interactions with humidity were never significant in the models for the prevalence of positive grid or density of larval habitats per grid.

**Figure 6:**
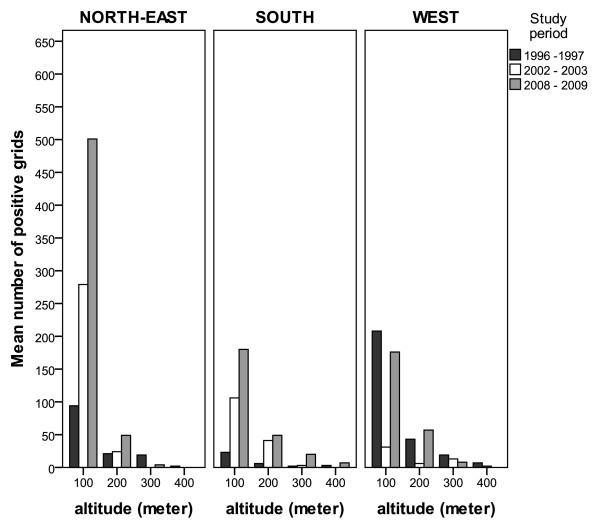
**The influence of altitude on the temporal distribution of *An. arabiensis *breeding sites in three distinct geographic areas in La Reunion Island**. The altitudinal distribution is presented by sections of 100 meters altitude until the altitude of 400 meters in each zone. Bars show the mean number of breeding habitat in sentinel grid cells within each study zone.

After removing spatial scale trend effects (by pooling data from the three zones) and controlling for variation in mean monthly temperature and rainfall (by including them in the GLM models), an additional effect of altitude (F_1, 13106 _= 368.04, *p *< 0.001), household density (F_1, 13106 _= 8.05, *p *= 0.005) but not its annual variations (year × household density interaction: F_2, 13106 _= 0.62, p = 0.53) on the number of breeding site per positive grid was found. In the Figure 6, the altitudinal distribution of positive larval habitats within each zone is presented according to the following gradients < 100 meters, 100-200, 200-300, 300-400. The spatial distributions, especially at local scales, of *Anopheles *breeding sites showed a decreasing trend for year-adjusted breeding site densities with increasing altitude up to 400 km. In addition, the average numbers of breeding sites per positive grid in area with low household density was consistently larger than that observed in area with higher household density (more than 200 premises per square kilometre, derived from GIS). Irrespective of the study zone, this increase was noticeable from the extent of 60.9 m to 600 meters far from humans. Overall, these results suggest that the numbers of breeding site per sampling grid would be expected to differ in different parts of a geographic scale due to the influence of local environmental characteristics, in addition to climatic factors.

## Discussion

Multi-year trend analyses of historical records of permissive grid cells wherein breeding sites were recorded highlighted a quantitative increase of functional breeding sites from 1996 to 2009. In addition, the retreat of their distribution range to lower altitudinal zones and the upward shift in the most remote littoral area in the north-eastern, south-western geographic regions suggests the possible influence of biogeographic factors, land-use and farming, climatic conditions, and control measures in recent decades.

First, in La Reunion Island mountain ranges and elevation greater than 500 m above sea level seemed to constitute barriers to the spread of *An. arabiensis *mosquitoes. This feature actually delimits the potential ecological zone where breeding sites occur, whereas on the African continent and Madagascar, this species is permanently present above 1,000 meters [19,20]. Secondly, the existence of torrents or ravines wetland, all subjected to periodic flooding and drying, may have been especially useful in intercepting and limiting *An. arabiensis *dispersal. It has been established that *Anopheles *mosquitoes can disperse over considerable distances but only go as far as is necessary to find hosts or suitable larval habitats [21,22]. Therefore, the availability of these topographic features, perhaps in addition to low rates of migration of adult *An. arabiensis *populations, might be responsible for the current patchy distribution of their larval habitats within the respective studied zones. Thirdly, La Reunion has experienced some improvements in average socio-economic status of the population over time [23,24]. Independently of larval control operations, this could have contributed to the observed reduction in habitats that can be exploited by *An. arabiensis *in the three studied zones.

During field collections of the data reported here, operation activities using *Bti *took place on a monthly basis to control *Anopheles *larvae in different aquatic habitats within each study zone and where they could be reached by the preventive teams. Each functional larval habitat found during the field surveys was treated at least on one occasion during the study period (JSD, pers. comm.). However, limitations of access into the ravines and other physical barriers have prevented the widespread insecticide applications within some aquatic habitats. The control foci - mainly targeted at the elected niches for the *An. arabiensis *(Table 2) - only produced short-lived results (at any level of coverage and habitat dynamics) and required regular interventions [9,25]. Previous vector control efforts using mass *Bacillus*-based larviciding efforts have been cited as effective in reducing mosquito populations [26]. Thus, it is likely that the observed reduction in the geographic distribution range of breeding sites could be attributed to the effect of larval suppressions with the *Bti *sprayed on surface water as implemented during the study period. Noteworthy, vector control against *An. arabiensis *was strongly decreased or relaxed during the chikungunya epidemic outbreak in 2006, so that in 2008 positive breeding habitats were found beyond their usual distribution range. This indicates the importance of the larval control to slow down the expansion of the vector.

The analyses of the occurrence data from 2006 to 2009 indicated restriction of the breeding site occurrence to three large geographic regions (Figure 2). This apparent patchy distribution of *An. arabiensis *breeding site coincides with the complete genetic heterogeneity observed among *An. arabiensis *populations from all localities of the Island [27]. It is likely that the reported genetic composition of these populations may have been driven by both the recent geographical discontinuity of suitable habitats colonized by *An. arabiensis *[28], and bottlenecks due to contemporary and historic influences of large-scale larval control pressure. However, the reduction in distribution due to control operations may have been minimized by meteorological influences in the southern; western and north-eastern zones during the whole study period.

The proportions of positive grids and the number of breeding sites were significantly higher in north-eastern than in the southern and western zones. The observed variations between the three zones underline the importance of the marked gradient in climatic factors in the dynamics aquatic stage of *Anopheles *mosquitoes. In addition, our data on climatic parameters (rainfall, temperature) reflect this diversity, so as the aggregated breeding habitat distributions. The absence of active breeding sites at some surveyed grid cells during the field surveys may have been a consequence of the decline in water availability and/or of the absence of recent oviposition owing to fluctuations in water 'quality' [29-31]. Alternatively, their occurrence may have also varied in space as a result of random appearance and disappearance of suitable aquatic habitats during seasonal succession [32-34]. Differences in rain water accumulations may be partially responsible for the seasonal fluctuations in the proportion of positive habitats. However, in the western zone the rainfall deficit, accentuated by the nature of the soil, often imposes to the farmers the recourse to watering by sprinkling in the orchards and gardens and to irrigation in sugar cane fields. These irrigated lands could contribute in maintaining vector populations year-round in this more arid western ecosystem. This would imply a greater adaptation of *An. arabiensis *mosquitoes to breeding sites of temporary nature than in both the western and north-eastern zones where open aquatic habitats (e.g. rock pools, roadside ditches, car tracks) frequently fed by heavy rain and water from more permanent source (e.g. river fringes) allow continuous breeding.

## Conclusion

On the basis of information collected in this study, functional breeding spots for *An. arabiensis *persisted in the respective study zones on La Reunion despite the continuous larval control effort over the past decades. The present report elicits several implications. First, *An. arabiensis *population distribution has probably shifted to ecologically suitable zones along the south-west and north east axis and persists in vast area in which water availability fluctuates with the season and year. This observation mitigates against the effectiveness of larval control programs that rely mainly on the use of microbial larvicides. The importance of temporal variation in environmental conditions will need to be recognized in future risk assessment and planning of larval control activities at broad geographical stratification of the island. Secondly, the continuous decrease in the geographic distribution range of this potential malaria vector within discrete environmental foci provides the opportunity to optimize control by directing and focusing control efforts at distinct areas. At present, when scientific communities spelt instructions for malaria eradication [35,36], integrated control strategies in La Reunion need to follow an area-wide campaign that allied tools against both larval and adult stages. Because of the obvious limitations of the larval control approach in the recent years, there is currently much interest in exploring new methods, such as Sterile Insect Techniques (SIT). The pattern distribution of malaria vectors in La Reunion is a good example to challenge the idea that the reduction of vector population through conventional methods is a prerequisite to the deployment of SIT [37]. However, in light of the above observations, further investigations on the patchy distribution and metapopulation structure of *An. arabiensis *in an evolving heterogeneous environment would be necessary in order to optimize future release of sterile males in a SIT area-wide operation.

## Competing interests

The authors declare that they have no competing interests.

## Authors' contributions

JSD and RG initiated the study, made data available in collaboration with the National Meteorological Agency and the National Geographic Institute. GLC undertook statistical analysis and drafted the manuscript. JSD made major contributions to the statistical analysis. All authors contributed to the writing of the manuscript and approved the submitted version of the manuscript.
